# Defining major trauma: a Delphi study

**DOI:** 10.1186/s13049-021-00870-w

**Published:** 2021-05-10

**Authors:** Lee Thompson, Michael Hill, Fiona Lecky, Gary Shaw

**Affiliations:** 1 North East Ambulance Service NHS Foundation Trust, Ambulance HQ, Bernicia House, Goldcrest Way, Newburn Riverside, Newcastle Upon Tyne, NE15 8NY England; 2 Northumbria University, Coach Lane Campus, Coach Lane, Newcastle Upon Tyne, NE7 7TR England; 3 University of Sheffield, Western Bank, Sheffield, S10 2TN England; 4 University of Manchester, Oxford Rd, Manchester, M13 9PL England; 5 Salford Royal Hospitals NHS Foundation Trust, Stott Lane, Salford, M6 8HD England; 6 Trauma Audit and Research Network, Summerfield House, 544 Eccles New Road, Salford, M5 5AP England

**Keywords:** Major trauma, Delphi, Prehospital

## Abstract

**Introduction:**

Retrospective trauma scores are often used to categorise trauma, however, they have little utility in the prehospital or hyper-acute setting and do not define major trauma to non-specialists. This study employed a Delphi process in order to gauge degrees of consensus/disagreement amongst expert panel members to define major trauma.

**Method:**

A two round modified Delphi technique was used to explore subject-expert consensus and identify variables to define major trauma through systematically collating questionnaire responses.

After initial descriptive analysis of variables, Kruskal-Wallis tests were used to determine statistically significant differences (*p* < 0.05) in response to the Delphi statements between professional groups. A hierarchical cluster analysis was undertaken to identify patterns of similarity/difference of response.

A grounded theory approach to qualitative analysis of data allowed for potentially multiple iterations of the Delphi process to be influenced by identified themes.

**Results:**

Of 55 expert panel members invited to participate, round 1 had 43 participants (Doctor *n* = 20, Paramedic *n* = 20, Nurse *n* = 5, other *n* = 2). No consistent patterns of opinion emerged with regards to professional group. Cluster analysis identified three patterns of similar responses and coded as trauma minimisers, the middle ground and the risk averse. Round 2 had 35 respondents with minimum change in opinion between rounds.

Consensus of > 70% was achieved on many variables which included the identification of life/limb threatening injuries, deranged physiology, need for intensive care interventions and that extremes of age need special consideration. It was also acknowledged that retrospective injury severity scoring has a role to play but is not the only method of defining major trauma. Various factors had a majority of agreement/disagreement but did not meet the pre-set criteria of 70% agreement. These included the topics of burns, spinal immobilisation and whether a major trauma centre is the only place where major trauma can be managed.

**Conclusion:**

Based upon the output of this Delphi study, major trauma may be defined as: “Significant injury or injuries that have potential to be life-threatening or life-changing sustained from either high energy mechanisms or low energy mechanisms in those rendered vulnerable by extremes of age”.

**Supplementary Information:**

The online version contains supplementary material available at 10.1186/s13049-021-00870-w.

## Background

**Fig. 1:**
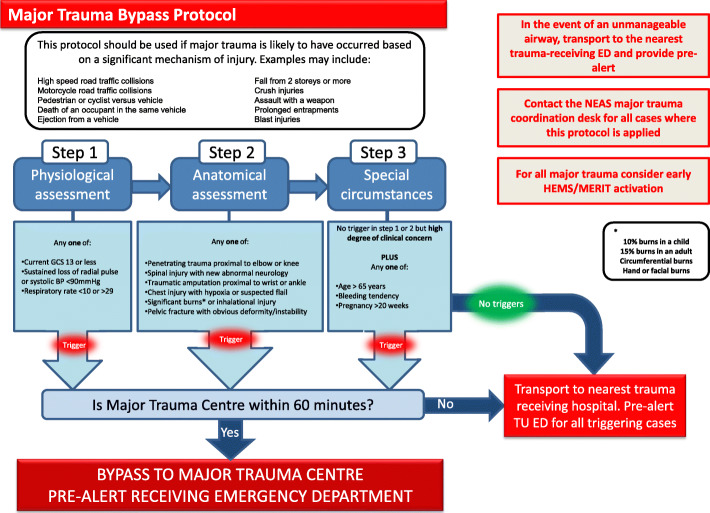
Major Trauma Triage Tool

Deaths in older children through to middle age include suicide, injury and poisoning as the main causes [1] and for those aged over 20 years injury is the most common cause of death for women aged 10 to 30 years and for men aged 15 to 35 years [2]. However, major trauma remains a relatively rare cause of death within England and Wales. In Scotland paramedic exposure to trauma accounts for 0.3% of case volume [3]. The lack of exposure to major trauma can cause anxiety and our perception of what is classified as major trauma is potentially complex. Within the UK major trauma triage tools similar to that shown in Fig. 1 assists prehospital clinicians to identify major trauma patients who may be suitable for primary transfer to specialist care at a Major Trauma Centre. The triage tool highlighted in Fig. 1 uses physiological elements that are present in other triage tools such as the Simple Triage and Rapid Treatment (START) triage tool and Revised Trauma Score (RTS), all of which can be used in the prehospital phase of care. The START tool is primarily for multiple casualty/major incident /disaster events and can be used by both medical and non-medical personal for rapid triage [4] although it does have a high incidence of over triage [5]. The RTS also has its limitations and it is not as sensitive to predicting outcomes (as does the tool highlighted in Fig. 1) [6]. Essentially these tools are used for triage purposes and are limited in their ability to define major trauma. However, they are commonly used within established trauma networks to identify trauma patients who need immediate management.

Although high energy mechanisms are often associated with major trauma, Kehoe, Smith et al. [7] have challenged this assumption. They highlight the change in major trauma patient groups who are now more elderly and have significant injuries with high injury severity scores (ISS) as a result of low energy mechanisms such as a fall from standing height.

There have been several publications that explore the definition of polytrauma, most notably those by Butcher and Balogh [8–11]. Their initial work highlighted the need for a consensus for the definition of polytrauma which progresses to the development of a definition that looks at retrospective scores as well as physiological parameters. Although potentially linked to the definitions of polytrauma, the definition of major trauma may differ. A literature review [12] identified that the most common definition for major trauma is a retrospective ISS of > 15. The injury severity score is an aggregation of the main injuries from each body region. All injuries receive a code which is generated from the Abbreviated Injury Scale (AIS) dictionary and each body region is scored 1–6 and then squared and the three highest scores added together. These scores have little utility in the prehospital and hyper-acute settings as accurate AIS codes are only generated after hospital imaging is completed [13]. They also do not fully describe major trauma to the non-specialist. With a lack of a descriptive definition [14] and in the absence of ISS, it is important to be able to define major trauma and provide context to prehospital clinicians, emergency medicine clinicians and non-specialists.

This study employed a Delphi process in order to gauge the degrees of consensus and disagreement amongst expert panel members, their views and definition of major trauma.

The research question assumed an exploratory focus:

“Which factors do subject experts and current prehospital care practitioners identify in defining major trauma in the absence of injury severity scores?”

The specific aims of the study included:

- To distil subject expert opinion concerning the definition of major trauma; and,
- To critically explore the extent of consensus in the definition of major trauma in the absence of ISS.

## Methods

### Study design and setting

A two round modified Delphi technique (with a potential third) was employed in order to explore subject-expert consensus and identify in-situ use of variables to define major trauma in the absence of ISS. This is facilitated through systematically collecting, analysing, coding and presenting questionnaire responses to the original expert panel participants with the explicit instruction to reflect upon their own individual responses in light of the collective group response. Participants are then invited to submit a revised response to the questionnaire should they wish to do so. Collectively this process might be referred to as one of iteration with controlled feedback. However, Scheibe et al. [15] identified that when faced with collective group responses, individual respondents have three options: to ignore the feedback, to maintain their views or to adapt a more extreme response to that originally expressed. Using experimental methods, these authors contended that the means by which the feedback is presented has the potential to introduce a distorting influence into the Delphi process in a way which is both difficult to predict and control for. The initial questionnaire can be found in supplementary material 1 along with the anonymised feedback in supplementary material 2.

The exploratory nature of the Delphi study allowed for feedback to be provided to the expert panel using group responses. To prevent any bias, and to ensure rigor throughout the process, all responses were anonymised and sent to all participants prior to undertaking round two with clear and precise instructions on how to manage the data and respond [16]. The feedback combined all the results of round 1 as simple graphs to illustrate all responses as well as a summary of the free text used throughout the questionnaire which summarised individual definitions of major trauma (see supplementary material 2). This was believed to provide new information that may generate new perspectives to achieve a group consensus.

The survey was designed to reflect the outcomes of a literature review [12] and the output from three focus groups [17] the results and conclusions of which are in supplementary materials 3 and 4. This included the domains:

- clinician factors, such as experience and exposure;
- patient factors, such as physiology, outcome measures and pre-trauma factors; and,
- situational factors, such as mechanism of injury.

Questions were designed around the domains highlighted above and included variables from both the literature review and focus groups (supplementary materials 3 and 4) in order to ascertain potential clustering factors including both observable (e.g. profession, experience and age) and unobservable factors (e.g. values, attitudes, opinions and preferences). Although the domains were known to the authors, these were not explicitly labelled within the survey instrument and therefore may not have been immediately apparent to participants. The questionnaire for subsequent rounds were intentionally unchanged from the initial questionnaire to aid analysis and to compare any significant changes in responses after the feedback had been provided to the participants. As such only minor amendments were made for clarification and to correct any inconsistencies, grammar and spellings.

Grant, Booth [18] recommend that the Delphi process should conclude after predetermined multiple iterations or when consistency between rounds is stable with unchanging opinion.

### Definition of consensus

Mubarak, Hatah [19] highlight that 100% agreement can seldom be achieved among experts and that an arbitrary percentage should be set prior to undertaking the study. Within our Delphi design, Likert type scales were used which give the option of a neutral response. With this in mind the research team set the arbitrary percentage of 70% agreement (positive or negative) as subject-expert consensus where the neutral score was not considered. The exception to this would be if the group agreement was more than or equal to a 70% neutral response. The main issue with including a mid-point/neutral option is that it becomes an easy option when the other options are potentially socially undesirable/controversial. Within the study design, whilst omitting the mid-point/neutral option was contemplated, it was ruled out on the basis that this may have led to further undesirable consequences. The initial concept of omitting a mid-point/neutral option would force the participant to choose the theoretical nearest positive or negative response from the neutral option. Chyung, Roberts [20] explored literature that concluded that when the neutral option is removed from Likert type scales the responses are distributed to the nearest alternative option but, many respondents simply did not respond leaving that question unanswered. With this in mind, several questions presented themselves for a simple binary response which may partially mitigate any neutral response. Dolnicar and Grün [21] highlight that this method provides an acceptable alternative to ordinal scales that may also improve the efficiency of the questionnaire.

### Sampling of study participants (expert panel)

The expert panel members, who will be referred to as participants within this study, were from a broad range of professional groups who are exposed to and manage major trauma patients within their everyday workplace. The use of the term ‘expert’ is commonplace in the lexicon of Delphi methodology and literature but does not imply expert status in the vernacular sense: It simply implies that panel members are purposively selected on the basis of a privileged knowledge base or experience. In this instance, panel members were purposively selected based upon diversity of experience and expertise within a single trauma network. Weinstein [22] explains there are two kinds of expertise: expertise in knowing (epistemic expertise) and expertise in doing (performance expertise). Bourne, Kole [23] explore the potentially abstract concept of expertise within elitism and cite exemplar individuals who are undoubtedly experts within their own domain and ‘one of a kind’. However, they also acknowledge the expert who is such due to their accumulation of hard work as well as ability. One of the strengths (and limitations) of this study was to capture the views of participants who were experts by virtue of their understanding and hard work at the patient interface within a single trauma network. Within the context of this study the expert panel were required to have first-hand experience of the hyper-acute trauma setting to which a definition of major trauma can be applied.

Whilst there are no absolute guidelines as to the number of participants that may contribute to the Delphi process [24], the aim was to have at least three individuals from each relevant professional group within the Northern Trauma Network (NTN) which covers the North East and Cumbria areas of England.

### Data collection and management

Ethical approval was granted through Integrated Research Application System (IRAS project ID: 237977).

We utilised a Delphi method with two iterations of questionnaires (with a potential third which was not required). The survey was conducted using the online system SurveyMonkey Inc. (San Mateo, California, USA). Panel members remained anonymous to one another throughout the data collection and analysis process. The Delphi study commenced on 12 December 2018 and ran through to 5 November 2019 (this time frame is discussed within the study limitations).

All data collected were stored electronically in a secure and password protected folder and anonymised prior to analysis.

### Validity and reliability

Sackman [25] suggested that the Delphi processes fail to meet standards of reliability and validity ‘*normally set for scientific methods*.’ However, careful scrutiny of Sackman’s assertions reveal that his concerns relate more to the methodological shortcomings of particular studies rather than overall methodological approach per se.

Anonymised results are believed to prevent attrition of panel members who may have a minority opinion [26] and minimises bias that certain individuals may create as well as contributing to the overall rigor of the study [16]. A short pilot study was carried out to refine the wording of the survey instruments and to remove potential ambiguities and ensure reliability of responses. All responses were anonymised and peer reviewed prior to any analysis and sharing with the panel members at repeated iterations between survey iterations.

### Data analysis

All quantitative data analysis was undertaken using the Statistical Package for the Social Sciences (SPSS; Version 26, IBM Inc.; Armonk, NY, USA). The level of statistically significance was predetermined as a *p* value of < 0.05 [27].

After initial descriptive analysis of variables, Kruskal-Wallis tests were used to determine statistically significant differences (*p* < 0.05) in response to the Delphi statements between the professional groups within the sample e.g. Doctors, Paramedics, Nurses and others which included managers, academics and administrators. The term ‘other’ was used to prevent unique individuals within specialised professional groups from being easily identified.

The Kruskal–Wallis test is a statistical method for ascertaining the significance of differences between the median values for *K+* sub-groups from within the same sample sometimes referred to as ‘ANOVA by Ranks’: this is the test of choice when analysing ordinal data such as that generated by the Delphi instrument.

No consistent patterns of opinion emerged in relation to professional group membership (Doctor / Paramedic / Nurse / other). The statistical parameters for the use of Kruskal Wallis suggest a minimum group membership of 5 [28]. Whilst the ‘other’ group failed to meet this parameter (*n* = 2), there was no theoretical basis to combine this group with any other.

Because no consistent patterns of difference emerged based upon professional group membership, a hierarchical cluster analysis was undertaken in order to identify patterns of similarity and difference of response within the data. Yim and Ramdeen [29] identified that ‘*Cluster analysis refers to a class of data reduction methods used for sorting cases, observations, or variables of a given dataset into homogeneous groups that differ from each other.*’ Cases (individual participants) are clustered based upon chosen characteristics – in this instance, similarity in the way they scored selected Delphi statements – *and NOT their professional grouping*. Cases in each specific cluster share many characteristics but are also dissimilar to those not belonging to that cluster. A three-cluster solution provided membership in each group of a size that would allow for further meaningful statistical comparison in order to determine qualitative differences in response patterns between the clusters. This was calculated using Ward’s method and squared Euclidian distance as a means to determine cluster membership whilst minimising variance within each cluster.

Therefore, in the current study, the cluster membership was based upon similarity in response to the Delphi statements. Arranging response patterns together and classifying these as belonging to different broader groups provides a means of applying some organisation to individual Delphi responses, which at first sight might appear highly individualised or even chaotic. The technique of cluster analysis originated in biology and ecology [30] and although the technique has been reasonably widely employed in social science analysis, it has not (to date) gained the same level of application in health research.

Free text data generated by questionnaire responses were managed and analysed using NVivo qualitative data analysis software, QRS International Pty Ltd., Version 11, 2015. Data were coded and reviewed to identify emerging themes [31].

A grounded theory approach to qualitative analysis of the free text data allowed for potentially multiple iterations of the Delphi process to be influenced by the generated data and themes identified. This inductive approach allowed for theoretical insights to be generated as the process was undertaken rather than testing preconceived hypotheses [32]. Within the context of this study it allowed for a thematic framework to distil variables into their most common denominators to provide generalisable themes appropriate to both the expert and layperson. This is not to imply *statistical* generalisation, but rather the type of qualitative *moderatum* generalisation identified by Williams [33].

## Results

**Fig. 2:**
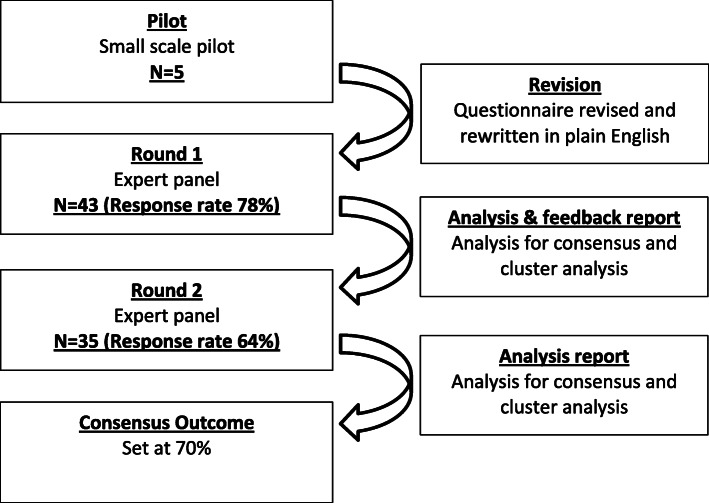
Study Process

Figure 2 highlights the Delphi study process and the frequency of responses throughout.

A text version of the questionnaire can be found in supplementary material 1. The results of round 1 which were used as feedback to the expert panel/participants can be found in supplementary material 2.

Table 1 describes the frequency of responding participants professional group alongside experience in years, including range and mean.

**Table 1:** Delphi participants by professional group and experience

Professional group Round 1 (n)	Experience in years Range (mean)*
Doctor (20)	6–21+ (14)
Paramedic (16)	6–20 (14)
Nurse (5)	0–21+ (12)
Other (2)	Not recorded
Total (43)	0–21+ (13)

*Rounded

Due to the level of expertise within very specific professional disciplines which specialise in major trauma, participants were placed into generic professional groups to prevent identifying individuals and potential bias. These groups were used within the context of the cluster analysis to identify differences between specific group responses. Table 2 highlights the response rates to each round of the study by professional group.

**Table 2:** Delphi participants by professional group and response rates

Invited to participate by professional group (n)	Round 1 n (Response %)	Round 2 n (Response %)
Doctor (20)	20 (100)	14 (70)
Paramedic (20)	16 (80)	16 (80)
Nurse (10)	5 (50)	3 (30)
Other (5)	2 (40)	2 (40)
Total (55)	43 (78)	35 (64)

### Round 1

Because no consistent patterns of opinion emerged in relation to professional group membership (Doctor / Paramedic / Nurse), a cluster analysis was performed in order to identify patterns of similarity of response within the data (whilst ignoring whether responses were made by professional group). Participants who did not complete all sections of the questionnaire (*n* = 7) were excluded from the cluster analysis.

Three distinctive clusters were identified and their composition by professional group is outlined in Table 3.

**Table 3:** Composition of clusters

Cluster	N (%)	Composition (%)
1	9 (25)	4 Doctors (44) 4 Paramedics (44) 1 Nurse (11)
2	20 (56)	10 Doctors (50) 7 Paramedics (35) 3 Nurses (15)
3	7 (19)	5 Doctors (71) 1 Paramedic (14) 1 Nurse (14)
		19 Doctors (53)
Total	36 (100)	12 Paramedics (33)
		5 Nurses (14)

Clusters 2 and 3 were very closely linked together and all clusters produced a normal distribution pattern.

Cluster 1 were coded as “Trauma Minimisers” owing to their answers indicating a high threshold for identifying major trauma. In relative terms, from a given number of trauma patients, cluster 1 participants would identify a very low percentage as major trauma.

Cluster 2 were coded as “The Middle Ground”. This cluster represented the majority of the Delphi participants as well as their respective professional groups. Cluster 2 identified what would be considered an appropriate proportion of major trauma based upon existing criteria seen in Fig. 1.

Cluster 3 were coded as “Risk Averse” as their answers indicated a very low threshold for identifying major trauma. From a given number of trauma patients cluster 3 would identify a high percentage as major trauma.

Table 4 highlights the areas of consensus within the first round Delphi questionnaire that was predetermined as > 70% agreement (see supplementary materials 1 and 2 for questionnaire and participant feedback/responses).

**Table 4:** Consensus on variables (Delphi round 1)

Variable	Consensus (**>** 70%)	%
Actual injuries^a^	Yes	100 (>med)
Only high energy mechanisms should be considered	Yes	97.5 (>disagree)
Physiology^a^	Yes	97.44 (>med)
Need for blood products^a^	Yes	92.3 (>med)
Age (> 65 years) special consideration^a^	Yes	89.75 (>med)
Experienced clinicians are able to identify major trauma patients	Yes	89.74 (>agree)
Need for ventilatory support^a^	Yes	89.47 (>med)
Intoxication makes triage difficult	Yes	87.5 (>agree)
Age (paediatric)^a^	Yes	87.18 (>med)
Age has no relevance	Yes	85 (>disagree)
Low energy mechanisms should be considered	Yes	85 (>Agree)
Elderly require different assessment/management	Yes	85 (>agree)
Need for surgical intervention^a^	Yes	84.61 (>med)
Triage tools always identify major trauma	Yes	82.5 (>disagree)
Mechanism of injury (MOI)^a^	Yes	82.5 (>med)
Scoring systems are the only way to identify major trauma	Yes	76.92 (>disagree)
Paediatrics require different assessment/management	Yes	77.5 (>agree)
Identified by clinical assessment (as opposed to mechanism of injury)	Yes	77.5 (>agree)
Can only be defined by retrospective scores	Yes	75 (>disagree)
Perceived need for Intensive Care Unit admission	Yes	75 (>agree)
Triage tools can identify patients who would benefit from MTC care	Yes	75 (>agree)
Outcome measures (e.g. injury severity scores)^a^	Yes	71.8 (>med)
Pre-existing frailty should be considered	Yes	70 (>agree)
Need for tranexamic acid (TXA)^a^	No	69.22 (>med) 30.77 (Low)
Need for pelvic binding^a^	No	64.1 (>med) 35.9 (low)
Perceived need for surgical intervention	No	62.5 (>agree) 22.5 (neutral) 15 (Disagree)
Major trauma can only be managed at an MTC	No	62.5 (>disagree) 15 (neutral) 22.5 (agree)
Need for spinal immobilisation^a^	No	61.54 (low) 38.47 (>med)
Clinicians high index of suspicion can identify major trauma without imaging	No	60 (>agree) 15 (neutral) 25 (disagree)
Burns should have a separate protocol	No	57.9 (>agree) 26.32 Neutral) 15.79 (disagree)
Previous medical history^a^	No	56.41 (low) 43.59 (med)
Burns should be included in major trauma triage	No	55.27 (>agree) 7.89 (Neutral) 36.85 (disagree)
Pre-existing co-morbidity should be considered	No	51.28 (>agree) 25.64 (neutral) 23.08 (disagree)

^a^Refers to multi-variable choice within question 1 (see supplementary material 1)

A single question within the survey instrument (question 23 - supplementary material 1) presented participants with a list of factors that could be acknowledged as the main variables in defining major trauma. This list was distilled from a comprehensive review of existing literature and earlier focus group research with practitioners. Respondents were asked to identify factors they viewed as relevant with a binary “yes” / “no” answer. Table 5 highlights the key variables from that list that achieved consensus from the Delphi participants.

**Table 5:** Key variables highlighted by participants in round 1

Variable identified	Consensus (**>** 70%)	%
Life threatening injuries	Yes	95
Limb threatening	Yes	92.5
Major blood loss	Yes	87.5
Suspected abdominal injury with haemodynamic instability	Yes	80
Injury causing reduced consciousness	Yes	72.5

There was an obvious consensus on many of the variables highlighted above as being of definitive major trauma. There were also some statistically significant variations in agreement between clusters in other variables (level of significance set as *p* < 0.05). These variations in agreement are described in Table 6.

**Table 6:** Variables where significant variation in agreement differs between clusters

Variable	Difference between clusters (C)			***p*** Value*
	Cluster	Differs from	Cluster	
**Identifier for major trauma**				
Need for spinal immobilisation	1	Differs from	2	< 0.01
Need for pelvic binding	1	Differs from	2 & 3	0.01
**Age has no relevance within major trauma**	3	Differs from	1 & 2	0.01
**Burns**				
Should be inc. within major trauma triage tool	3	Differs from	1 & 2	< 0.01
Burns should have a separate protocol	1	Differs from	2 & 3	< 0.01
**Defining major trauma**				
Pre-existing frailty should be considered	1	Differs from	2 & 3	< 0.01
Pre-existing comorbidities should be considered	1	Differs from	2	< 0.01

* *p* value rounded to 2 decimal places (Independent samples Kruskal-Wallis test)

Free text responses within the questionnaire were coded and subject to thematic analysis. Questions 21 and 22 (see supplementary material 1) asked for free text descriptions of the participants’ personal and, if appropriate, work place definitions of major trauma. This emergent grounded theory analysis allowed for subtle adjustment to the survey instrument prior to iteration 2 of the Delphi process. Table 7 provides a summary overview of these coded themes.

**Table 7:** Frequency of variables highlighted in qualitative analysis of free text

Variable	Round 1 n
Significant injury/Polytrauma	24
Life threatening/changing/disability	18
Mechanism of Injury (MOI)	14
Specialist input	12
Physiological changes	10
Prolonged treatment/Rehab	8
Age	6
Previous medical conditions	3
Bespoke/patient specific care	2
ISS	1
Total number of variables	98

### Round 2

Of the original 43 respondents from the first round, 35 participants completed the second round of the Delphi. Several members had since left their original place of work and were unable to be contacted.

The survey instrument utilised in round 2 remained relatively unchanged from the initial instrument used in round 1 i.e. the structure of the instrument did not change at all and subtle wording changes were influenced by participants’ prior qualitative responses. This was intentional to aid analysis and to compare any significant changes in response due to the feedback provided in supplementary material 2. Non-parametric related-samples Wilcoxon Signed rank test was utilised to analyse difference in responses with the significance level set at < 0.05.

There were only modest changes in overall responses between iterations 1 and 2. Five statements moved from non-consensus to consensus status, with only a single statement moving from consensus to non-consensus (see Table 8). None of the consensus changes (summarised in Table 8) proved statistically significant in their own right but their combined effect was sufficient to alter the overall consensus. A single statistically significant change was ‘major trauma patients can only be managed at an MTC’ which, although statistically significant it still did not meet the agreed consensus level of 70% and therefore did not change its overall status.

**Table 8:** Changes in consensus between rounds 1 and 2 (questions 1–20)

Variable	Consensus (**>** 70%)	Round 1%	Round 2%	Related Samples Wilcoxon Signed Rank Test
Pre-existing frailty should be considered	Changed to No	70 (>agree)	63.64 (>agree) 21.21 (neutral) 15.15 (>disagree)	0.142
Need for tranexamic acid (TXA)*	Changed to Yes	69.22 (>med) 30.77 (Low)	79.41 (>med)	0.124
Need for pelvic binding*	Changed to Yes	64.1 (>med) 35.9 (low)	76.47 (>med)	0.432
Perceived need for surgical intervention*	Changed to Yes	62.5 (>agree) 22.5 (neutral) 15 (Disagree)	70.59 (>agree)	0.218
Clinicians high index of suspicion can identify major trauma without imaging	Changed to Yes	60 (>agree) 15 (neutral) 25 (disagree)	70.59 (>agree)	0.084
Burns should have a separate protocol	Changed to Yes	57.9 (>agree) 26.32 (neutral) 15.79 (disagree)	76.47 (>agree)	0.325

The single change from consensus to non-consensus concerned the statement ‘Injury causing reduced consciousness’ which moved from a 72.5% agreement to a below consensus agreement of 65%. This variable was one of multiple options that a participant could choose from to help support their definition of major trauma (question 23 in supplementary material 5). The results of Delphi results were distilled further as part of a reductive strategy (grounded theory/thematic analysis) to provide an elegant and generalisable definition of major trauma. Table 9 highlights the themes produced by the results of Tables 4, 5 and 7.

**Table 9:** Factors identified as definitive components of major trauma

Reductive Coding	Table 4 Variables	Table 5 Variables	Table 7 Variables
Potentially Life Threatening	Deranged physiology	Life threatening injuries	Life threatening injuries
		Suspected abdominal injury with haemodynamic instability	Physiological changes
		Injury causing reduced consciousness	
	Need for blood products	Major blood loss	
	Need for ventilatory support		
	Potential need for ICU		Specialist input required
Potentially Life Changing	Need for surgical intervention	Limb threatening injuries	Life changing injuries
			Significant injury/polytrauma
			Prolonged treatment/rehabilitation
Other	Actual injuries		
	Clinical experience/skills/perception		
	MOI (high and low energy)		
	Age (paediatrics and older adults		
	Frailty		
	Interventions (TXA, Pelvic binding)		
	ISS/scoring/triage		

## Discussion

### Statement of principle findings

Abersek [34] explains the concept of elegance within science as the distilling of potentially infinite complexity, which can be interpreted by many as dull and mundane, into seemingly simple answer. This distilled complexity conceptualises the topic into its simplest form to express the essence of the issue, which can provide a potent yet elegant solution. It is worth noting that elegance within science does not detract from the complex nature of scientific endeavour but articulates that complexity in a deep and meaningful way which is often viewed as simple. The thematic analysis highlighted in Table 8 visualises this process may be an over simplification of our definition of major trauma. However, it does have at its very foundation the generalisable building blocks to defining major trauma that can be applied to all from expert to non-specialist/layperson alike. There are nuances in every field of practice and, as such, these foundations can be built upon to make generalisable concepts specific to individuals or professional groups by the addition of individual/professional group idiosyncrasies. An insightful comment by one participant highlighted this concern with regards to definitions needing context depending upon area of practice, ‘*How you define it will be based on where in the patient journey that patient is. End [diagnosis] after 3 weeks in hospital with access to complex imaging and specialist input is different to how it will be at the ED front door or in the prehospital setting’.*

The areas of consensus highlighted in Table 5 were replicated throughout the study in the free text as well as reflecting the association with other key variables highlighted within the results and summarised in Table 9. They highlight that life and limb threatening injuries are without doubt the variables that define major trauma. Included within that table are major blood loss, abdominal injury with haemodynamic instability and reduced consciousness which could be addressed under deranged physiology. Deranged physiology could also be argued to highlight life and limb threatening injuries. It was also noted that only using high energy mechanisms should be discounted. This is reflected in the work by Magnone, Ghirardi [35], Potter, Kehoe [36] and Stuke, Duchesne [37] who highlight that, in isolation, mechanism of injury does not correlate well with outcomes.

The participants within this study do not significantly change their opinions between rounds with the exception of those highlighted above. Furthermore, during the cluster analysis there was no clear difference in response between individual disciplines and each cluster had an even distribution of professional groups.

In the main, consensus was achieved in many variables highlighted within the study. Within round 1 several aspects did not meet the agreed consensus level such as the need for TXA, pelvic binding and ‘potential need’ for surgical intervention (as opposed to actual need) but in round 2 responses provided a shift in agreement and these variables consequently met the agreed 70% consensus. As such they may be considered as surrogate markers of major trauma and applied as a consequence of the potential underlying injury.

Although two burns-related statements were presented to the participants, a non-consensus reaching majority in iteration 1 (which became a consensus agreement after iteration 2) and paradoxically asserted that burns should have a separate protocol from the major trauma triage tool and yet also be included in the major trauma triage tool. These conflicting statements may be due to the wording and placement of the statements within the instrument, but other than this no strong conclusions can be drawn from this change in consensus status.

Again, the majority, but not meeting the prespecified consensus level, disagree that major trauma can only be managed at an MTC. This may reflect the views of the regional specialists that are distributed throughout the trauma units or that sub-groups of patients may be best managed locally.

A low percentage of agreement on whether to consider comorbidities and previous medical history in identifying potential major trauma may be reflective of the composition of the participants within the Delphi study. Owing to the nature of the research topic, in the context of defining major trauma in the hyper-acute phase of care, there was an obvious lack of participants from the rehabilitation and long-term care disciplines. These sub-acute disciplines may have an alternative perspective with regards to the variables that should be considered in defining major trauma.

It is perhaps reassuring and a testament to the specialist/expert participants that a patients actual injuries are a primary focus in identifying major trauma and also based on that patients individual circumstances. A bespoke model for identifying major trauma should take into account the unique nature of an individual patients episode of care that includes their age and expected physiology and that not all mechanisms are equal based on an individual’s unique response. It is also noted that experts within the hyper-acute trauma setting do not agree with triage tools and scoring systems being able to identify all major trauma. This may reflect the wealth of experience and exposure to major trauma within the participant group and a common theme that ran through the study was that major trauma is unique to the individual at that time where injury/injuries threaten life or limb.

### Strengths and weaknesses of the study

The Delphi study provided a technique to gain consensus on defining major trauma by the experts within that specialist area across disciplines. Delphi techniques have previously been used in order to seek expert consensus in prehospital care matters [38–40]. However, Delphi methodology has been subject to criticism on the basis of methodological flaws, most notably: sampling and use of ‘experts’; anonymity; and the issue of enforced consensus [24]. Throughout the study the authors remained cognisant of these criticisms during the design phase of this study. The title of expert is also very subjective and relies upon the context within which supposed expertise lies. Within the context of this study it was a conscious decision to use experts with current lived experiences of working predominantly within the trauma setting in a hands on clinical context. This may be considered both a strength and weakness of the study and the regional specific expertise may produce its own idiosyncrasies.

There was a significant drop out rate between both rounds (round 1 *n* = 43, round 2 *n* = 35), however, this is not uncommon in relation to repeated administrations of the same survey. The drop out rate may partially be contributed to the long-time frame over which the study was conducted. Unfortunately, the two lead researchers had family members with acute illness and consequential bereavement which had a significant impact on the overall timeframes that could not be avoided.

Within the cluster analysis it was difficult to provide an existing criteria in which to compare the differences between the ‘trauma minimisers’, ‘the middle ground’ and ‘risk averse’ groups as we had yet to provide a definition of major trauma. As such the potential criteria were to use ISS as an outcome score or those who would be positively identified by the major trauma triage tool (Fig. 1). Both of which have their own limitations but as a pragmatic and surrogate marker the regional major trauma triage tool was used as it could contextualise the responses of the participants who all practiced within the region.

The regional trauma network and the individuals who work within it are a very close community. There may be a risk of unintentional homogenous thinking due to the isolated nature and familiarity within the group. There is also a risk of excluding the views and perceptions of those who are not specialists or who work in the sub-acute disciplines within the region although it is believed that the definition of major trauma will be transferable and generalisable within all settings. It is an intentionally broad definition in its application to provide an elegant solution from a complex process to allow it to be appropriate to all. However, each professional group may have their own idiosyncrasies and therefore additional criteria may be added to their own specific definition of major trauma which would then exclude other groups. As a general definition it stands alone but is also enhanced by the addition of discipline specific variables which complement their unique definition of major trauma.

### Strength and weaknesses in relation to other studies, discussing important differences in results

The authors are unaware of any prior consensus study which has attempted to define major trauma in the absence of ISS or other scoring mechanisms (although there are examples that relate to defining polytrauma [10] and prehospital tools that explore triage such as START [4] and RTS [6]). It is therefore difficult to compare this study to other studies or literature.

### Meaning of the study

This Delphi study highlights the group consensus of the expert panel to the definition of major trauma in the hyper-acute setting. It was interesting that although clusters were created (trauma minimisers, the middle ground and the risk averse) there was no real difference in composition within those clusters highlighting that differences were not based on profession. It is hoped the concluding definition can provide a reference for non-specialists, academics and/or clinicians where retrospective scoring systems provide little context or meaning.

### Unanswered questions and future research

The definition of major trauma from this Delphi study is partly subjective and therefore open to interpretation. ISS or other scoring systems provide an objective measure but have very limited utility within the hyper-acute setting. Future research may be able to identify objective measures that consider the principles within this study.

## Conclusions

Based upon the previous literature review, focus groups and the output of this Delphi study, major trauma may be defined as: “Significant injury or injuries that have potential to be life-threatening or life-changing sustained from either high or low energy mechanisms especially in those rendered vulnerable by extremes of age”. This simple, single sentence definition is a concise solution which can be complimented by additional criteria to make it specific for various professional groups or to reflect the patients position within their overall journey of care.

### Supplementary Information

**Additional file 1: Supplementary material 1.** Delphi study survey round 1.

**Additional file 2: Supplementary material 2.** Delphi study survey results/feedback round 1.

**Additional file 3: Supplementary material 3.** Outcomes of literature review.

**Additional file 4: Supplementary material 4.** Outcomes of focus groups to define major trauma.

**Additional file 5: Supplementary material 5.** Results of delphi study survey round 2.

## Data Availability

The data generated for the Delphi study is available upon reasonable request to the lead author.

## Appendix 2

### Personal definitions of Major Trauma

**Participant 1:** *Injuries sustained in a traumatic way (external forces acting on the body). Major trauma would include significant injuries especially to trunk of body (rather than isolated limb) and injuries resulting in significant physiological changes. Significant trauma usually results in need for blood transfusion, surgical management, involves multiple body systems/areas.*

**Participant 2:** *These are very much just thoughts, but - Major trauma signifies altered physiology and disruption/injuries likely to disrupt one of the ‘significant’ body systems -* i.e. *respiratory/circulatory/neurological. However there are then subtleties with special patient populations -* i.e. *the elderly, and specific injury patterns* e.g. *burns. It would be useful to have specific triage tools for these populations. I don’t think mechanism alone should be considered.*

**Participant 3:** *Patient physiology on scene and throughout the patient journey. Not to consider M.O.I.*

**Participant 4:** *Major trauma, to me, is significant traumatic injury which is potentially life threatening or life changing and which will require a prolonged hospital treatment, recovery and physiotherapy. Important factors include rapidly identifying life threatening injuries and fixing what can be fixed with rapid transport. Less important factors at the acute pre hospital stage include; pre-existing medical conditions and some observations like BM and tympanic temperature.*

**Participant 5:** N/A.

**Participant 6:** N/A.

**Participant 7:** *A [patient] that has suffered multiple injuries.*

**Participant 9:** *Major Trauma is a collective term for high velocity mechanism. High injury severity scores are a retrospective marker which can be achieved from low velocity mechanisms especially in older patients. Despite the fact that older patients may suffer significant injury from low velocity mechanisms, that does not mean transfer to MTC from scene (or at all) is necessary - AGGRESSIVE CONSERVATIVE MANAGEMENT ie recognise injuries early, treat pain properly, keep hydrated, attempt early mobilisation, monitor nutrition and bowel, look after kidney function - this is what most frail trauma patients need and this can be delivered in TU and MTC.*

**Participant 10:** *A patient who presents with severe injuries that require medical or surgical intervention to treat them. This can include patients with a high energy mechanism or pattern of injury that raises concerns as well as those with co-morbidities that raise the likelihood of injury.*

**Participant 11:** *Multi-system injury with deranged physiology.*

**Participant 12:** *More than one injury to limbs or organs in the body from external forces that will significantly disrupt auto regulation/ ability to complete basic motor function without considerable assistance or intervention from specialities. Significant enough harm that the individuals normal functioning is severely effected regardless of age. Requires care in a hospital setting for longer than 3 days.*

**Participant 13:** *Injuries which pose an immediate risk to life, cause long term disability or those that prevent a patient returning to their baseline level of function.*

**Participant 14:** *An injury pattern that has the potential to result in death or significant morbidity for the affected patient.*

**Participant 15:** *Deranged physiological following a traumatic insult where identified or suspected injuries require or have the potential to require critical life-saving interventions. If it is not life or limb threatening it is not major trauma. In the absence of the above it is simply ‘trauma’ and should therefore be managed as such.*

**Participant 16:** *Major Trauma are injuries which are life threatening where a delay in the patient getting to a hospital that can safely treat the patient with a co-ordinated response would be detrimental to the patient.*

**Participant 17:** *Life threatening injuries with high likelihood of prolonged disability.*

**Participant 18:** *High impact RTC fall from height fall from standing in height in > 65.*

**Participant 19:** *life threatening, multiple limb injury, abdominal / chest wounds, major haemorrhage, extensive burns.*

**Participant 20:** *Major trauma is a traumatic injury sufficient to cause a significant physiological insult; the exact types of injury will vary across age groups and frailty but major trauma in all cases is associated with worse outcomes and increased mortality.*

**Participant 21:** *I would identify major trauma as a mechanism that results in multiple or life threatening injuries. This can be mechanisms of high or low energy.*

**Participant 22:** *Life threatening or life changing trauma. Defined by injuries that are found or strongly suspected, and deranged physiology that fit those findings / suspicions. Poorly predicted and defined by mechanism.*

**Participant 23:** *That requiring specialist input.*

**Participant 24:** *Any significant multi system illness with a traumatic cause.*

**Participant 25**: *A condition where the patients injury burden is more than their physiological reserves and may require multiple system support.*

**Participant 26:** *Significant injury from a traumatic event that requires specialist or multi-disciplinary intervention (including professions allied to medicine input* e.g. *specialist physiotherapy).*

**Participant 27:** *MT is an injury sustained by a significant MOI causing traumatic injuries that will impact on patient and cause a high ISS.*

**Participant 28:** N/A.

**Participant 29:** *Pre hospital major trauma should be about which patients will benefit from the added value a major trauma centre brings.*

**Participant 30:** N/A.

**Participant 31:** *Major trauma should be split into suspected MT, current assessment of MT and Definition of MT. MT = Significant injury that requires definitive clinical care at a specialist centre, following any mechanism that puts the patients life or multi-limb at risk. Remote assessment for dispatch should be an experienced clinicians personal feelings following the gathering of subjective and objective information from scene. Assessment should reflect the objective information being presented to you at scene. Treatments should reflect assessment and the definition of Major Trauma should then and only then be stated following clinician assessment.*

**Participant 32:** *An injury of sufficient severity to require urgent specialist interventions. To consider - patients age, resp rate, gcs, area/type of injury, bp (radial pulse?). Not to consider - mechanism, feel there should be no mention to this at all.*

**Participant 33:** *Any patient with significant polytrauma involving one or more systems that may need specialist intervention.*

**Participant 34:** *Definition of Major Trauma is an accumulation or constellation of injuries which are potentially life threatening or changing. This would be classically called polytrauma. Major Trauma could include isolated injuries high enough in severity to meet the criteria above such as severe head injuries I don’t believe mechanism is helpful due to changes in demographics the relevance of severe poly/major trauma from low energy transfer has increased and requires specialist care.*

**Participant 35:** *Anatomical injuries that, if not managed in a timely fashion, will inevitably result in deranged physiology and lead to significant morbidity/mortality.*

**Participant 36:** N/A.

**Participant 37:** *Significant injuries resulting in death or disability if not appropriately managed.*

**Participant 38:** *How you define it will be based on where in the patient journey that patient is. End dx after 3 weeks in hospital with access to complex imaging and specialist input is different to how it will be at the ED front door on in the pre-hospital setting.*

**Participant 39:** *The physiological impact of trauma and the requirement for physiological support eg ventilation, blood products and surgical intervention represent trauma that has caused greatest injury and deviation from normal physiological status. This would incorporate the physiological effect of extremes age and premorbid condition.*

**Participant 40:** *A complex, multisystem pathological state arising as a result of injury (rather than illness) which left untreated will progress to multi organ failure and death.*

**Participant 41:** *For me it’s about the need for urgent critical care interventions PHEA/ blood transfusion/ pleural drainage or early access to DCS (Damage control surgery).*

**Participant 42:** *Potential life limiting injuries caused by non-natural events (RTC, assault, falls), causing major injuries/disability to the patient.*

***Participant 43:*** *Patient with multiple, complex or significant injuries that has the potential to cause prolonged recovery, disability or death. Sustained from a blunt or penetrating mechanism*

## Appendix 3

### Work base definitions of Major Trauma

**Major trauma bypass protocol (× 9 respondents)** +.

No specific definition although the Bypass protocol is used with some clinician experience if bypass is not met, but still suspect MTC required.

We have a major trauma tool which identifies patients for bypass to an MTC, this does not necessarily mean they are all major trauma.

**ISS > 15 (× 8 respondents)** +.

It reflects the current guidance of ISS. Agree that this retrospective scoring makes life difficult particularly in the acute phase to highlight those requiring specialist trauma care. Need to consider potential major trauma due to high prevalence of “stealth trauma” injuries.

It utilises a significant mechanism of injury (with some examples, but none exhaustive list), plus altered physiology or significant anatomical injury or high degree of clinical concern.

We do have a major trauma bypass tool which is used to determine if a patient is eligible for a MTC or normal A&E department. No definitive practice to determine a yes or no answer to ‘is this major trauma’ just clinical judgement and experience alongside the bypass tool.

In truth I am not entirely sure. I do however know that my area of practice interacts with more than one NHS ambulance service. Anecdotally I have observed that the term ‘major trauma’ is used more frequently, and at a much lower threshold in one service, than in another.

Trauma resulting in multiple injuries and need for admission.

MOI, physiology and special circumstances.

Major trauma is any injury that has the potential to cause prolonged disability or death.

Significant mechanism Anatomical and physiological changes Injuries including head, chest, abdomen, pelvis and multiple limb injuries.
