# Supplementary material for: Defining major trauma: a Delphi study
Source: Scand J Trauma Resusc Emerg Med. 2021 May 10;29:63. doi: 10.1186/s13049-021-00870-w (PMC8108467; doi:10.1186/s13049-021-00870-w)
Supplement: Supplementary file 1 — Additional file 1: Supplementary material 1. Delphi study survey round 1. [file 13049_2021_870_MOESM1_ESM.docx]

# Supplementary material 1

## **DELPHI STUDY SURVEY ROUND 1**

1. From the options below rate the items importance with regards to its role in defining major trauma

|  | This factor should not be considered | Low importance | Medium importance | High importance | This is the only factor to consider |
| --- | --- | --- | --- | --- | --- |
| Mechanism of injury |  |  |  |  |  |
| Actual injuries sustained |  |  |  |  |  |
| Physiology (e.g. Glasgow Coma Score, Respiration Rate, Systolic Blood Pressure) |  |  |  |  |  |
| Age (Paediatric) |  |  |  |  |  |
| Age (>65 years) |  |  |  |  |  |
| Previous medical history |  |  |  |  |  |
| Outcome measures such as Injury Severity Scores (ISS) |  |  |  |  |  |
| Need for surgical intervention |  |  |  |  |  |
| Need for ventilatory support |  |  |  |  |  |
| Need for blood products |  |  |  |  |  |
| Need for Tranexamic Acid (TXA) |  |  |  |  |  |
| Need for spinal immobilisation (eg. Collar, Blocks, Scoop) |  |  |  |  |  |
| Need for pelvic binding/splinting |  |  |  |  |  |
| Other: please specify below |  |  |  |  |  |

| Other: |  |
| --- | --- |

1. Major trauma should be identified by a clinical assessment and actual/perceived injury pattern regardless of the Mechanism of Injury (e.g. High energy v Low energy)

| Strongly Disagree | Disagree | Neither Agree or Disagree | Agree | Strongly Agree |
| --- | --- | --- | --- | --- |
|  |  |  |  |  |

1. The older trauma patient (aged 65 years +) should be assessed/triaged/managed differently compared to younger adult trauma patients

| Strongly Disagree | Disagree | Neither Agree or Disagree | Agree | Strongly Agree |
| --- | --- | --- | --- | --- |
|  |  |  |  |  |

1. Paediatric trauma patients (aged less than 12 years) should be assessed/triaged/managed differently to adult trauma patients

| Strongly Disagree | Disagree | Neither Agree or Disagree | Agree | Strongly Agree |
| --- | --- | --- | --- | --- |
|  |  |  |  |  |

1. Age has no relevance in trauma triage

| Strongly Disagree | Disagree | Neither Agree or Disagree | Agree | Strongly Agree |
| --- | --- | --- | --- | --- |
|  |  |  |  |  |

1. Burns (in the non-polytrauma patient):

|  | Strongly Disagree | Disagree | Neither Agree or Disagree | Agree | Strongly Agree |
| --- | --- | --- | --- | --- | --- |
| Burns should be included within the major trauma triage |  |  |  |  |  |
| Burns should have a separate protocol/triage from Major Trauma |  |  |  |  |  |

1. Pre-existing frailty should be considered when identifying major trauma

| Strongly Disagree | Disagree | Neither Agree or Disagree | Agree | Strongly Agree |
| --- | --- | --- | --- | --- |
|  |  |  |  |  |

1. Pre-existing medical conditions (co-morbidities) should be considered when identifying major trauma

| Strongly Disagree | Disagree | Neither Agree or Disagree | Agree | Strongly Agree |
| --- | --- | --- | --- | --- |
|  |  |  |  |  |

1. Major trauma can only be defined retrospectively using Injury Severity Scores

| Strongly Disagree | Disagree | Neither Agree or Disagree | Agree | Strongly Agree |
| --- | --- | --- | --- | --- |
|  |  |  |  |  |

1. Scoring systems are the only way to identify major trauma

| Strongly Disagree | Disagree | Neither Agree or Disagree | Agree | Strongly Agree |
| --- | --- | --- | --- | --- |
|  |  |  |  |  |

1. Experienced clinicians are able to identify major trauma patients

| Strongly Disagree | Disagree | Neither Agree or Disagree | Agree | Strongly Agree |
| --- | --- | --- | --- | --- |
|  |  |  |  |  |

1. Only high energy mechanisms of injury should be considered in identifying major trauma

| Strongly Disagree | Disagree | Neither Agree or Disagree | Agree | Strongly Agree |
| --- | --- | --- | --- | --- |
|  |  |  |  |  |

1. Major trauma triage tools always identify major trauma patients

| Strongly Disagree | Disagree | Neither Agree or Disagree | Agree | Strongly Agree |
| --- | --- | --- | --- | --- |
|  |  |  |  |  |

1. Low energy mechanisms of injury (such as a fall from standing) should be considered within major trauma if injury pattern suggests significant injury

| Strongly Disagree | Disagree | Neither Agree or Disagree | Agree | Strongly Agree |
| --- | --- | --- | --- | --- |
|  |  |  |  |  |

1. Intoxicated patients make triage of major trauma difficult

| Strongly Disagree | Disagree | Neither Agree or Disagree | Agree | Strongly Agree |
| --- | --- | --- | --- | --- |
|  |  |  |  |  |

1. A clinician with a high index of suspicion can confidently identify major trauma without specialist imaging.

| Strongly Disagree | Disagree | Neither Agree or Disagree | Agree | Strongly Agree |
| --- | --- | --- | --- | --- |
|  |  |  |  |  |

1. A perceived need for ongoing surgical intervention can be a key factor in identifying major trauma

| Strongly Disagree | Disagree | Neither Agree or Disagree | Agree | Strongly Agree |
| --- | --- | --- | --- | --- |
|  |  |  |  |  |

1. A perceived need for Intensive Care Unit (ITU) admission can be a key factor in identifying major trauma

| Strongly Disagree | Disagree | Neither Agree or Disagree | Agree | Strongly Agree |
| --- | --- | --- | --- | --- |
|  |  |  |  |  |

1. Major Trauma Bypass Protocols identify patients who would benefit from definitive care at a Major Trauma Centre and not just patients with high Injury Severity Scores.

| Strongly Disagree | Disagree | Neither Agree or Disagree | Agree | Strongly Agree |
| --- | --- | --- | --- | --- |
|  |  |  |  |  |

1. Major Trauma patients can only be managed at a Major Trauma Centre (excluding Traumatic Cardiac Arrest, compromised airway, transport time greater than 60 minutes).

| Strongly Disagree | Disagree | Neither Agree or Disagree | Agree | Strongly Agree |
| --- | --- | --- | --- | --- |
|  |  |  |  |  |

1. How would you personally define 'Major Trauma' in your own words? Please include factors you think should be considered and those you think should not be considered

|  |
| --- |

1. Does your area of practice have a definition for Major Trauma and if so how do they define Major Trauma?

|  |
| --- |

1. Please tick the variables you would use to define Major Trauma

| Life threatening injuries |  | Major Trauma is dependent on multiple factors that are unique to the individual patient at a given time |  |
| --- | --- | --- | --- |
| Limb threatening injuries |  | Injury Severity Score (>15) |  |
| Any injury that requires specialist intervention |  | Injury causing new neurology |  |
| Major blood loss |  | Suspected spinal injury requiring immobilisation |  |
| Injury to more than one limb |  | Suspected abdominal injury causing haemodynamic instability |  |
| Burns greater than 15% (10% Child) |  | Suspected pelvic injury requiring splinting |  |
| Injury causing reduced consciousness |  | Other Please state below: |  |
| High energy mechanism (for example but not limited too: roll over RTC, Fall from >2m, Gun shot wound) |  | Free text: | |

**The questions on this page will help us identify emerging trends and themes within the context of the multi-disciplined approach to major trauma.**

**Confidentiality: No personal information will be collected and survey responses will be collated anonymously using an identifying number known only to the participant and lead investigator. Some background information such as area of practice, length of experience in practice and experience within Major Trauma. All responses received in the study will be strictly confidential, and your identity will not be divulged. Direct quotes to free-text answers may be used as part of the study report or later Delphi iterations or research area, but these will be not be traceable back to you.**

1. What is your area of practice with regards to trauma:

| HEMS Paramedic |  |
| --- | --- |
| HEMS Doctor |  |
| Paramedic |  |
| Emergency Medicine (Doctor) |  |
| Intensive Care (Doctor) |  |
| Surgical (Doctor) |  |
| Academic |  |
| Other (please specify below) |  |
| Free text: | |

1. Years experience (post qualification)

| 0-5 years |  |
| --- | --- |
| 6-10 years |  |
| 11-15 years |  |
| 16-20 years |  |
| 21 years + |  |

1. In which setting do you usually work

| Major Trauma Centre |  |
| --- | --- |
| Trauma Unit |  |
| Rehabilitation |  |
| Prehospital |  |
| Non-trauma status hospital |  |
| Academia |  |
| Administration |  |
| Other (please specify) |  |
| Free text: | |

**Thank you.**

That is the end of the questionnaire. Thank you for taking the time to participate. We will inform you of the findings and ask for your comment on the findings as soon as possible.
