# Supplementary material for: Defining major trauma: a Delphi study
Source: Scand J Trauma Resusc Emerg Med. 2021 May 10;29:63. doi: 10.1186/s13049-021-00870-w (PMC8108467; doi:10.1186/s13049-021-00870-w)
Supplement: Supplementary file 2 — Additional file 2: Supplementary material 2. Delphi study survey results/feedback round 1. [file 13049_2021_870_MOESM2_ESM.docx]

# Supplementary material 2. DELPHI STUDY SURVEY RESULTS/FEEDBACK ROUND 1

**Defining Major Trauma:**

**A Delphi Study.**

**Feedback: Delphi study round 1.**

Hello all and thank you for taking the time to complete the first round of the Delphi study into defining major trauma it was very much appreciated. The data took some time to analyse and produced some valuable outputs.

The Delphi technique seeks to obtain consensus on the opinions of experts through a series of structured questionnaires. As part of the process, the responses from each round are fed back in summarised form to the participants who are then given an opportunity to respond to the emerging data. The Delphi is therefore an iterative multi-stage process designed to combine opinion into group consensus on the variables that define major trauma.

Based on these outputs we would like you to digest the following data which may influence how you answer the second round of the Delphi Study. The questions themselves have remained relatively unchanged, however, the feedback from the first round is designed to provide you with information that may bring together a common understanding of major trauma and a working definition of major trauma.

Results:

There were three distinct clusters of participants whose answers were similar (clusters 2 and 3 were very closely linked together) and produced a normal distribution pattern.

Cluster 1 were coded as “Trauma Minimisers” owing to their answers indicating a high threshold for identifying major trauma. In relative terms, from a given number of trauma patients, cluster 1 participants would identify a **very low** percentage as major trauma.

Cluster 2 were coded as “The Middle Ground”. This cluster represented the majority of the Delphi participants as well as their respective professional groups. Cluster 2 identified what would be considered an appropriate proportion of major trauma based upon existing criteria.

Cluster 3 were coded as “Risk Averse” as their answers indicated a very low threshold for identifying major trauma. From a given number of trauma patients cluster 3 would identify a **high** percentage as major trauma.

Seven participants out of a total of 43 participants did not answer all the questions within the questionnaire and were excluded from the cluster analysis based on the limited availability of data.

*Table 1. Composition of clusters.*

| **Cluster** | **N (%)** | **Composition (%)** |
| --- | --- | --- |
| 1 | 9 (25) | - 4 Doctors (44) - 1 Nurse (1) - 4 Paramedics (44) |
| 2 | 20 (56) | - 10 Doctors (50) - 3 Nurses (15) - 7 Paramedics (35) |
| 3 | 7 (19) | - 5 Doctors (71) - 1 Nurse (14) - 1 Paramedic (14) |

There was an obvious consensus on many of the variables highlighted as defining major trauma. These patterns are clear in the graphs seen in appendix 1. There were however some statistically significant variations in agreement between clusters in other variables (level of significance set as p <0.05).

*Table 2. Variables where significant difference occurs between cluster opinions.*

| **Variable** | **Difference between clusters (C)** | | | **p Value*** |
| --- | --- | --- | --- | --- |
|  | Cluster | Differs from | Cluster |  |
| Need for spinal immobilisation (as an identifier for major trauma) | 1 | - | 2 | <0.01 |
| Need for pelvic binding (as an identifier for major trauma) | 1 | - | 2 & 3 | 0.01 |
| Age has no relevance within major trauma | 3 | - | 1 & 2 | 0.01 |
| Burns should be included within Major Trauma Triage Tool | 3 | - | 1 & 2 | <0.01 |
| Burns should have a separate protocol | 1 | - | 2 & 3 | <0.01 |
| Pre-existing frailty should be considered (when defining major trauma) | 1 | - | 2 & 3 | <0.01 |
| Pre-existing comorbidities should be considered (when defining major trauma) | 1 | - | 2 | <0.01 |

* p value rounded to 2 decimal places (Independent samples Kruskal-Wallis test).

We have included many graphs which represent the distribution of answers given to some of the questions asked within the first round of the Delphi study (appendix 1). We have also included the free text of your personal definitions of major trauma (appendix 2) and your work base definitions (appendix 3).

**What now?**

Please take your time to digest the information in this document and then click on the link below or scan the QR code with a smart phone to complete the questionnaire. It is almost exactly the same questionnaire as the first but your answers may now be slightly different or may not change at all.

Can I thank you once again for taking the time to read through this information and complete the questionnaire. Without your help this project would not be possible.


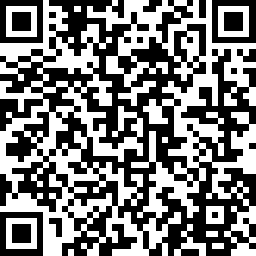
<https://www.surveymonkey.com/r/FP39ZGP>


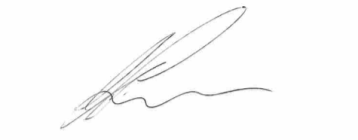
Best regards as always,

XXXX
