# Supplementary material for: Defining major trauma: a Delphi study
Source: Scand J Trauma Resusc Emerg Med. 2021 May 10;29:63. doi: 10.1186/s13049-021-00870-w (PMC8108467; doi:10.1186/s13049-021-00870-w)
Supplement: Supplementary file 3 — Additional file 3: Supplementary material 3. Outcomes of literature review. [file 13049_2021_870_MOESM3_ESM.docx]

# Supplementary material 3

## **OUTCOMES OF LITERATURE REVIEW**

(12)

Table 1. Summary of individual variables identified within the literature for defining major trauma

| **Criteria for defining major trauma** | ***(n)*** |
| --- | --- |
| Retrospective injury scores (inc. ISS, NISS, AIS, etc.) | 109 |
| Injury Severity Score (ISS) | 103 |
| Fatal outcome | 21 |
| Injury type/pattern | 16 |
| Intensive Care Unit (ICU) admission | 12 |
| Requires surgical intervention | 12 |
| Mechanism of injury (MOI) | 9 |
| Haematocrit decrease | 7 |
| Abbreviated Injury Scale (AIS) | 6 |
| New Injury Severity Score (NISS) | 6 |
| Requiring ventilation | 5 |
| Receiving blood products | 4 |
| Deranged physiology | 2 |
| Revised Trauma Score (RTS) | 2 |
| Trauma Injury Severity Score (TRISS) | 1 |
| International Classification of Diseases-derived ISS (ICISS) | 1 |
| Hospital Trauma Index ISS (HTI-ISS) | 1 |
| Paediatric Trauma Score (PTS) | 1 |
| Prehospital index greater than 3 | 1 |

Table 2. Potential prehospital variables identified as defining major trauma

| **Variables** | **(n)** |
| --- | --- |
| Fatal outcome | 21 |
| Injury type/pattern | 16 |
| Intensive Care Unit (ICU) admission | 12 |
| Requires surgical intervention | 12 |
| Mechanism of injury (MOI) | 9 |
| Haematocrit decrease | 7 |
| Requiring ventilation | 5 |
| Prehospital index | 4 |
| Receiving blood products | 4 |
| Deranged physiology | 2 |
| Revised Trauma Score (RTS) | 2 |
| Paediatric Trauma Score (PTS) | 1 |

Concluding statement within paper:

The most common definition of major trauma in contemporary and historical use is that of ‘an ISS greater than 15’. However, in the pre-hospital environment, retrospective scoring systems are not available and other variables must be considered. Based upon this review, a working definition of major trauma is suggested as: ‘A traumatic event resulting in fatal injury or significant injury with accompanying deranged physiology, regardless of MOI, and/or is predicted to require significant treatment sequelae such as ICU admission, surgical intervention, or the administration of blood products.’
