# Supplementary material for: Defining major trauma: a Delphi study
Source: Scand J Trauma Resusc Emerg Med. 2021 May 10;29:63. doi: 10.1186/s13049-021-00870-w (PMC8108467; doi:10.1186/s13049-021-00870-w)
Supplement: Supplementary file 4 — Additional file 4: Supplementary material 4. Outcomes of focus groups to define major trauma. [file 13049_2021_870_MOESM4_ESM.docx]

# Supplementary material 4. OUTCOMES OF FOCUS GROUPS TO DEFINE MAJOR TRAUMA

(17)

Table 1. Clinician factors in defining major trauma.

| Sub-theme | Factor |
| --- | --- |
| Experience | - Exposure to (or lack of) - Identifying injuries and ongoing care needs - Specific patient group needs - Intuition/instinct - Includes all the other themes below |
| Clinical Concern | - Linked to experience and potential injuries |
| Difficulties | - Communication issues - Environmental factors - Adrenaline rush (effecting decision making) - Distracting factors - Limited information |
| Index of suspicion | - Based on MOI and potential for injury |
| Potential for injury | - Suspicion based on experience and mechanism of injury and assessment |

Table 2. Patient factors in defining major trauma.

| Sub-theme | Factor |
| --- | --- |
| Physiology | - Altered physiology |
| Outcome measures | - Injuries - Life changing - Need for surgical intervention - Rehabilitation |
| Pre-trauma Factors | - Age - Previous medical history - Medications - Co-morbidities |

Table 3. Situation factors in defining Major Trauma.

| Sub-theme | Factors |
| --- | --- |
| Bespoke | Every patient, environment, situation is unique and requires a bespoke management plan |
| Mechanism of Injury | - Low energy - High energy - Influence of alcohol |
| Triage | - Tools have a role to play - Triage tools make generalisations but potentially miss many patients |

Concluding statement within paper:

Major trauma is unique to every provider, patient and situation that requires a bespoke management strategy. While MOI can raise the index of suspicion that major trauma has occurred, minor mechanisms, such as a fall from standing height, should not be discounted when identifying major trauma. There are challenges with accurately triaging patients at either end of the age spectrum, making the development of age-specific triage tools a focus for future research.

In the absence of retrospective scores, and based on using the data from the focus groups, we propose the following pre-hospital definition of major trauma: Any injury (or injuries) that have the potential to be life-threatening or life-changing,  including those sustained from low energy mechanisms in people rendered vulnerable by extremes of age, comorbidities or frailty, resulting in significant physiological compromise (haemodynamic instability, reduced consciousness, respiratory compromise) and/or significant anatomical abnormality that may require immediate intervention.
