# Supplementary material for: Defining major trauma: a Delphi study
Source: Scand J Trauma Resusc Emerg Med. 2021 May 10;29:63. doi: 10.1186/s13049-021-00870-w (PMC8108467; doi:10.1186/s13049-021-00870-w)
Supplement: Supplementary file 5 — Additional file 5: Supplementary material 5. Results of delphi study survey round 2. [file 13049_2021_870_MOESM5_ESM.docx]

Supplementary material 5. **RESULTS OF DELPHI STUDY SURVEY ROUND 2**

**Q 1. From the options below rate the items importance with regards to its role in defining major trauma**

|  | This factor should not be considered  n (%) | Low importance  n (%) | Medium importance  n (%) | High importance  n (%) | This is the only factor to consider  n (%) |
| --- | --- | --- | --- | --- | --- |
| Mechanism of injury | 0 | 6 (18.18) | 18 (54.55) | 8 (24.24) | 1 (3.03) |
| Actual injuries sustained | 0 | 0 | 1 (2.94) | 32 (94.12) | 1 (2.94) |
| Physiology (e.g. Glasgow Coma Score, Respiration Rate, Systolic Blood Pressure) | 0 | 0 | 6 (17.65) | 28 (83.35) | 0 |
| Age (Paediatric) | 0 | 3 (8.82) | 21 (61.76) | 10 (29.41) | 0 |
| Age (>65 years) | 0 | 3 (8.82) | 11 (32.35) | 20 (58.82) | 0 |
| Previous medical history | 2 (5.88) | 17 (50.00) | 11 (32.35) | 4 (11.76) | 0 |
| Outcome measures such as Injury Severity Scores (ISS) | 3 (9.09) | 7 (21.21) | 11 (33.33) | 11 (33.33) | 1 (3.03) |
| Need for surgical intervention | 2 (6.06) | 2 (6.06) | 9 (27.27) | 20 (60.61) | 0 |
| Need for ventilatory support | 2 (5.88) | 1 (2.94) | 5 (17.65) | 25 (73.53) | 0 |
| Need for blood products | 1 (2.94) | 1 (2.94) | 5 (14.71) | 27 (79.41) | 0 |
| Need for Tranexamic Acid (TXA) | 2 (5.88) | 5 (14.71) | 15 (44.12) | 12 (35.29) | 0 |
| Need for spinal immobilisation (eg. Collar, Blocks, Scoop) | 5 (14.71) | 15 (44.12) | 12 (35.29) | 2 (5.88) | 0 |
| Need for pelvic binding/splinting | 2 (5.88) | 6 (17.65) | 17 (50.00) | 9 (26.47) | 0 |
| Other: please specify below | 5 (55.56) | 2 (22.22) | 2 (22.22) | 0 | 0 |

| Other: |  |
| --- | --- |

**Q2. Major trauma should be identified by a clinical assessment and actual/perceived injury pattern regardless of the Mechanism of Injury (e.g. High energy v Low energy)**

| Strongly Disagree n (%) | Disagree  n (%) | Neither Agree or Disagree n (%) | Agree  n (%) | Strongly Agree  n (%) |
| --- | --- | --- | --- | --- |
| 1 (2.94) | 3 (8.82) | 0 | 21 (61.76) | 1. (26.47) |

**Q3. The older trauma patient (aged 65 years +) should be assessed/triaged/managed differently compared to younger adult trauma patients**

| Strongly Disagree n (%) | Disagree  n (%) | Neither Agree or Disagree n (%) | Agree  n (%) | Strongly Agree  n (%) |
| --- | --- | --- | --- | --- |
| 0 | 2 (5.88) | 2 (5.28) | 22 (64.71) | 1. (23.53) |

**Q4. Paediatric trauma patients (aged less than 12 years) should be assessed/triaged/managed differently to adult trauma patients**

| Strongly Disagree n (%) | Disagree  n (%) | Neither Agree or Disagree n (%) | Agree  n (%) | Strongly Agree  n (%) |
| --- | --- | --- | --- | --- |
| 0 | 3 (8.82) | 1 (2.94) | 23 (67.65) | 1. (20.59) |

**Q5. Age has no relevance in trauma triage**

| Strongly Disagree n (%) | Disagree  n (%) | Neither Agree or Disagree n (%) | Agree  n (%) | Strongly Agree  n (%) |
| --- | --- | --- | --- | --- |
| 10 (29.41) | 20 (58.82) | 3 (8.82) | 0 | 1. (2.94) |

**Q6. Burns (in the non-polytrauma patient):**

|  | Strongly Disagree n (%) | Disagree  n (%) | Neither Agree or Disagree n (%) | Agree  n (%) | Strongly Agree  n (%) |
| --- | --- | --- | --- | --- | --- |
| Burns should be included within the major trauma triage | 0 | 10 (31.25) | 4 (12.50) | 16 (50.00) | 3 (9.38) |
| Burns should have a separate protocol/triage from Major Trauma | 0 | 4 (11.76) | 4 (11.76) | 21 (61.76) | 1. (14.71) |

**Q7. Pre-existing frailty should be considered when identifying major trauma**

| Strongly Disagree n (%) | Disagree  n (%) | Neither Agree or Disagree n (%) | Agree  n (%) | Strongly Agree  n (%) |
| --- | --- | --- | --- | --- |
| 1 (3.03) | 4 (12.12) | 7 (21.21) | 17 (51.52) | 1. (12.12) |

**Q8. Pre-existing medical conditions (co-morbidities) should be considered when identifying major trauma**

| Strongly Disagree n (%) | Disagree  n (%) | Neither Agree or Disagree n (%) | Agree  n (%) | Strongly Agree  n (%) |
| --- | --- | --- | --- | --- |
| 1 (2.94) | 11 (32.35) | 9 (26.47) | 11 (32.35) | 1. (5.88) |

**Q9. Major trauma can only be defined retrospectively using Injury Severity Scores**

| Strongly Disagree n (%) | Disagree  n (%) | Neither Agree or Disagree n (%) | Agree  n (%) | Strongly Agree  n (%) |
| --- | --- | --- | --- | --- |
| 5 (15.63) | 21 (65.63) | 4 (12.50) | 2 (6.25) | 0 |

**Q10. Scoring systems are the only way to identify major trauma**

| Strongly Disagree n (%) | Disagree  n (%) | Neither Agree or Disagree n (%) | Agree  n (%) | Strongly Agree  n (%) |
| --- | --- | --- | --- | --- |
| 5 (15.63) | 23 (71.23) | 4 (12.50) | 0 | 0 |

**Q11. Experienced clinicians are able to identify major trauma patients**

| Strongly Disagree n (%) | Disagree  n (%) | Neither Agree or Disagree n (%) | Agree  n (%) | Strongly Agree  n (%) |
| --- | --- | --- | --- | --- |
| 0 | 1 (3.03) | 4 (12.12) | 21 (63.64) | 1. (21.21) |

**Q12. Only high energy mechanisms of injury should be considered in identifying major trauma**

| Strongly Disagree n (%) | Disagree  n (%) | Neither Agree or Disagree n (%) | Agree  n (%) | Strongly Agree  n (%) |
| --- | --- | --- | --- | --- |
| 7 (20.59) | 25 (73.53) | 1 (2.94) | 1 (2.94) | 0 |

**Q13. Major trauma triage tools always identify major trauma patients**

| Strongly Disagree n (%) | Disagree  n (%) | Neither Agree or Disagree n (%) | Agree  n (%) | Strongly Agree  n (%) |
| --- | --- | --- | --- | --- |
| 4 (11.76) | 22 (64.71) | 3 (8.82) | 1. (14.71) | 0 |

**Q14. Low energy mechanisms of injury (such as a fall from standing) should be considered within major trauma if injury pattern suggests significant injury**

| Strongly Disagree n (%) | Disagree  n (%) | Neither Agree or Disagree n (%) | Agree  n (%) | Strongly Agree  n (%) |
| --- | --- | --- | --- | --- |
| 1 (2.94) | 1 (2.94) | 0 | 27 (79.41) | 1. (14.71) |

**Q15. Intoxicated patients make triage of major trauma difficult**

| Strongly Disagree n (%) | Disagree  n (%) | Neither Agree or Disagree n (%) | Agree  n (%) | Strongly Agree  n (%) |
| --- | --- | --- | --- | --- |
| 0 | 0 | 3 (8.82) | 23 (67.65) | 1. (23.53) |

**Q16. A clinician with a high index of suspicion can confidently identify major trauma without specialist imaging.**

| Strongly Disagree n (%) | Disagree  n (%) | Neither Agree or Disagree n (%) | Agree  n (%) | Strongly Agree  n (%) |
| --- | --- | --- | --- | --- |
| 0 | 3 (8.82) | 7 (20.59) | 22 (64.71) | 1. (5.88) |

**Q17. A perceived need for ongoing surgical intervention can be a key factor in identifying major trauma**

| Strongly Disagree n (%) | Disagree  n (%) | Neither Agree or Disagree n (%) | Agree  n (%) | Strongly Agree  n (%) |
| --- | --- | --- | --- | --- |
| 0 | 4 (11.76) | 6 (17.65) | 23 (67.65) | 1. (2.94) |

**Q18. A perceived need for Intensive Care Unit (ITU) admission can be a key factor in identifying major trauma**

| Strongly Disagree n (%) | Disagree  n (%) | Neither Agree or Disagree n (%) | Agree  n (%) | Strongly Agree  n (%) |
| --- | --- | --- | --- | --- |
| 0 | 4 (11.76) | 6 (17.65) | 23 (67.65) | 1. (2.94) |

**Q 19. Major Trauma Bypass Protocols identify patients who would benefit from definitive care at a Major Trauma Centre and not just patients with high Injury Severity Scores.**

| Strongly Disagree n (%) | Disagree  n (%) | Neither Agree or Disagree n (%) | Agree  n (%) | Strongly Agree  n (%) |
| --- | --- | --- | --- | --- |
| 0 | 3 (9.09) | 4 (12.12) | 21 (63.64) | 1. (15.15) |

**Q20. Major Trauma patients can only be managed at a Major Trauma Centre (excluding Traumatic Cardiac Arrest, compromised airway, transport time greater than 60 minutes).**

| Strongly Disagree n (%) | Disagree  n (%) | Neither Agree or Disagree n (%) | Agree  n (%) | Strongly Agree  n (%) |
| --- | --- | --- | --- | --- |
| 2 (5.88) | 14 (41.18) | 7 (20.59) | 8 (23.53) | 3 (8.82) |

**Table 1. Consensus levels and changes between rounds 1 and 2 (questions 1 to 20).**

| **Variable** | **Consensus (>70%)** | **Round 1**  **%** | **Round 2**  **%** | **Wilcoxon Signed Rank test** |
| --- | --- | --- | --- | --- |
| Actual injuries* | Yes | 100 (>med) | 100 (>med) | 0.083 |
| Only high energy mechanisms should be considered | Yes | 97.5 (>disagree) | 94.12 (>disagree) | 0.132 |
| Physiology* | Yes | 97.44 (>med) | 100 (>med) | 1.000 |
| Need for blood products* | Yes | 92.3 (>med) | 94.12 (>med) | 1.000 |
| Age (>65 years) special consideration* | Yes | 89.75 (>med) | 91.17 (>med) | 0.462 |
| Experienced clinicians are able to identify major trauma patients | Yes | 89.74 (>agree) | 84.85 (>agree) | 0.527 |
| Need for ventilatory support* | Yes | 89.47 (>med) | 91.18 (>med) | 1.000 |
| Intoxication makes triage difficult | Yes | 87.5 (>agree) | 91.18 (>agree) | 1.000 |
| Age (paediatric)* | Yes | 87.18 (>med) | 91.17 (>med) | 0.805 |
| Age has no relevance | Yes | 85 (>disagree) | 88.23 (>disagree) |  |
| Low energy mechanisms should be considered | Yes | 85 (>agree) | 94.12 (>agree) | 0.796 |
| Elderly require different assessment/management | Yes | 85 (>agree) | 88.24 (>agree) |  |
| Need for surgical intervention* | Yes | 84.61 (>med) | 87.88 (>med) | 0.134 |
| Triage tools always identify major trauma | Yes | 82.5 (>disagree) | 76.47 (>disagree) | 0.057 |
| Mechanism of injury (MOI)* | Yes | 82.5 (>med) | 81.82 (>med) | 0.971 |
| Scoring systems are the only way to identify major trauma | Yes | 76.92 (>disagree) | 86.86 (>disagree) | 0.796 |
| Paediatrics require different assessment/management | Yes | 77.5 (>agree) | 88.24 (>agree) |  |
| Identified by clinical assessment (as opposed to mechanism of injury) | Yes | 77.5 (>agree) | 88.23 (>agree) |  |
| Can only be defined by retrospective scores | Yes | 75 (>disagree) | 81.26 (>disagree) | 0.971 |
| Perceived need for Intensive Care Unit admission | Yes | 75 (>agree) | 70.59 (>agree) | 0.808 |
| Triage tools can identify patients who would benefit from MTC care | Yes | 75 (>agree) | 78.79 (agree) | 0.541 |
| Outcome measures (e.g. injury severity scores)* | Yes | 71.8 (>med) | 69.69 (>med) | 0.921 |
| Pre-existing frailty should be considered | Yes/No | 70 (>agree) | 63.64 (>agree)  21.21 (neutral)  15.15 (>disagree) | 0.142 |
| Need for tranexamic acid (TXA)* | No/Yes | 69.22 (>med)  30.77 (Low) | 79.41 (>med) | 0.124 |
| Need for pelvic binding* | No/Yes | 64.1 (>med)  35.9 (low) | 76.47 (>med) | 0.432 |
| Perceived need for surgical intervention* | No/Yes | 62.5 (>agree)  22.5 (neutral)  15 (Disagree) | 70.59 (>agree) | 0.218 |
| Major trauma can only be managed at an MTC | No | 62.5 (>disagree)  15 (neutral)  22.5 (agree) | 47.06 (>disagree)  20.59 (neutral)  32.35 (>disagree) | 0.041 |
| Need for spinal immobilisation | No | 61.54 (low)  38.47 (>med) | 58.83 (<low)  41.17 (>med) | 0.499 |
| Clinicians high index of suspicion can identify major trauma without imaging | No/Yes | 60 (>agree)  15 (neutral)  25 (disagree) | 70.59 (>agree) | 0.084 |
| Burns should have a separate protocol | No/Yes | 57.9 (>agree)  26.32 (neutral)  15.79 (disagree) | 76.47 (>agree) | 0.325 |
| Previous medical history | No | 56.41 (low)  43.59 (med) | 55.88 (<low)  44.11 (>med) | 0.830 |
| Burns should be included in major trauma triage | No | 55.27 (>agree)  7.89 (Neutral)  36.85 (disagree) | 59.38 (>agree)  12.5 (neutral)  31.25 (disagree) | 0.533 |
| Pre-existing co-morbidity should be considered | No | 51.28 (>agree)  25.64 (neutral)  23.08 (disagree) | 38.23 (>agree)  26.47 (neutral)  35.29 (>disagree) | 0.148 |

*Refers to multi-variable choice within question 1.

**Table 2. Changes in consensus between rounds 1 and 2 (questions 1-20)**

| **Variable** | **Consensus (>70%)** | **Round 1**  **%** | **Round 2**  **%** |
| --- | --- | --- | --- |
| Pre-existing frailty should be considered | Changed to No | 70 (>agree) | 63.64 (>agree)  21.21 (neutral)  15.15 (>disagree) |
| Need for tranexamic acid (TXA)* | Changed to Yes | 69.22 (>med)  30.77 (Low) | 79.41 (>med) |
| Need for pelvic binding* | Changed to Yes | 64.1 (>med)  35.9 (low) | 76.47 (>med) |
| Perceived need for surgical intervention* | Changed to Yes | 62.5 (>agree)  22.5 (neutral)  15 (Disagree) | 70.59 (>agree) |
| Clinicians high index of suspicion can identify major trauma without imaging | Changed to Yes | 60 (>agree)  15 (neutral)  25 (disagree) | 70.59 (>agree) |
| Burns should have a separate protocol | Changed to Yes | 57.9 (>agree)  26.32 (neutral)  15.79 (disagree) | 76.47 (>agree) |

**Q21. How would you personally define 'Major Trauma' in your own words? Please include factors you think should be considered and those you think should not be considered.**

Major trauma is any injury that has the potential to cause life changing or life threatening injuries.

Each case on its own value, usually done by examination and identification of suspected injury, the use of trauma triage tools can be misleading if not applied correctly.

The increased potential of prolonged disability or death

An injury pattern that presents with abnormal physiology or level of consciousness and is consistent with a high energy or dangerous mechanism.

A patient who has sustained significant injuries that may be life or limb threatening and benefit from extended levels of care both in the prehospital environment and within hospital.

Injury or injuries that pose a risk to life, cause long term disability or prevent the patient returning to their baseline level of function. Mechanism of injury, physiology, pre-morbid function and comorbidities should be considered alongside suspected and identified injuries when considering the likely impact on the patient.

A patient who has sustained significant injuries or who may have experience significant forces with a high degree of clinical concern to their age.

Severe or life threatening injuries

I think of Major trauma as a mechanism based descriptor. Injury severity scores denote Injury severity and severe injuries can come from low velocity (non Major trauma ) mechanisms. To my mind the term Major trauma describes a high velocity mechanism with a high risk of severe multisystem injury with or without haemodynamic instability and which will benefit from being met by a trauma team in hospital who see these patients more frequently.

Major Trauma is traumatic injury significant enough to pose a threat to life either from isolated severe injury or constellation of injuries (polytrauma)

Life threatening or life changing injuries to that particular patient

Multisystem or complex single system injuries, causing physiological derrangement, and requiring specialist medical input

Multiple injuries - polytrauma MOI consistent with high suspicion of injury Requirement of multi - specialist team approach to patient care Elderly falls from standing - not major trauma but can have significant traumatic injuries

Trauma which significantly effects the normal functioning of the body and which requires specialist treatment and rehabilitation.

Where the actual injuries suspected or confirmed, alongside an indicative physiological response are such that there is a severe, imminent or immediate threat to life, regardless of the causative mechanism.

An injury or group of injuries sustained from external factors that threaten the integrity of the body’s main systems and their ability to function normally and sustain life or motion. Consideration should be given to influences that may effect, including type of insult, frailty of patient, capacity to self identify significant injuries and capacity to communicate

Significant injury to an individual that will have a major impact on their current physical/mental way of living

Major trauma is when the type and severity of injury is life threatening and potentially life limiting

Traumatic injuries that are potentially life-threatening or life-changing and result in altered physiology

Traumatic injuries sufficient enough to threaten life or limb. Injuries which may be life changing and involve a long recovery period

Major trauma is a pattern of injuries disrupting essential body functions. The injuries compromise either airway, breathing, circulation or neurological function. Mechanism of injury plays a part in defining it but is not the sole factor. There should definitely be amendments for elderly, with a lower threshold for suspecting significant injuries.

A traumatic insult to the patient resulting in multiple injuries which are life or limb threatening in the short term or have a significant impact on patient's lifestyle (in terms of morbidity and disability) in the longer term.

Trauma that results in physiological compromise and which needs immediate treatment to optimize physiology

Significant systemic injury as a result of a traumatic episode e.g. fall from standing in an elderly frail patient or potentially latent injuries in a child.

Major trauma is an injury that can cause a patient a potential prolonged disability or death.

Major traumatic injuries

Injuries with an immediate threat to life or risking long term morbidity

Major trauma is an umbrella term for conditions that would benefit from MTC input or where the patient would suffer for lack of MTC input. These are normally severe injuries to one system or injuries to multiple systems from an acute non-disease based process

Much along the lines of Major Incident - An injury pattern requiring physiological support and expert intervention outwith standard practice

**Table 3. Frequency of free text coding**

| Variable | Round 2 n |
| --- | --- |
| Significant injury/Polytrauma | 12 |
| Life threatening/changing/disability | 27 |
| Mechanism of Injury (MOI) | 9 |
| Specialist input | 5 |
| Physiological changes | 8 |
| Prolonged treatment/Rehab | 3 |
| Age | 4 |
| Previous medical conditions | 2 |
| Bespoke/patient specific care | 6 |
| ISS | 1 |
| Total number of variables | 77 |

**Table 4. Frequency of free text coding between rounds 1 and 2**

| Variable | Round 1 n | Round 2 n |
| --- | --- | --- |
| Significant injury/Polytrauma | 24 | 12 |
| Life threatening/changing/disability | 18 | 27 |
| Mechanism of Injury (MOI) | 14 | 9 |
| Specialist input | 12 | 5 |
| Physiological changes | 10 | 8 |
| Prolonged treatment/Rehab | 8 | 3 |
| Age | 6 | 4 |
| Previous medical conditions | 3 | 2 |
| Bespoke/patient specific care | 2 | 6 |
| ISS | 1 | 1 |
| Total number of variables | 96 | 77 |

**Q22. Does your area of practice have a definition for Major Trauma and if so how do they define Major Trauma?**

High mechanism of injury and ISS greater than 15

Yes, the increased potential of prolonged disability or death

Yes, they use the regional trauma triage tool.

Initially defined using major trauma bypass criteria. Retrospectively defined using ISS >15.

Unsure

Nothing agreed

mechanism of injury and primary survey as basis of care form trauma patients

As per the NTN guidelines

Not exactly. There is no 'written' definition. There is a nod to the local MTC bypass criteria however this is not referred to often. It is largely based on clinical presentation and the clinicians suspicion of injury pattern alongside the pts likely ongoing speciality requirements. We have discussed a retrospective audit on our specificity when compared to the local MTC bypass protocol in order to validate (or otherwise) our current practice.

Yes although the definitions used aren’t comparable as they don’t account for the differential locations of MTc to PT. Objectively biased to ensure over triage.

Follow local trauma bypass and focus on high mechanism of injury.

We have a major trauma bypass protocol which can be used to guide the clinician.

A significant mechanism of injury, plus any of abnormal physiological, anatomical assessment or special circumstances.

As defined in the Major Trauma Bypass Protocol

They do not

Same as NTN one

Defined by patients that meet the major trauma bypass tool

**Q23. Please tick the variables you would use to define Major Trauma**

**Table 3. Individual variable consensus**

| **ANSWER CHOICES** | **RESPONSES n (%)** |
| --- | --- |
| Life threatening injuries | 31 (91.18) |
| Limb threatening injuries | 30 (88.24) |
| Any injury that requires specialist intervention | 14 (41.18) |
| Major blood loss | 31 (91.18) |
| Injury to more than one limb | 7 (20.59) |
| Burns greater than 15% (10% Child) | 18 (52.94) |
| Injury causing reduced consciousness | 22 (64.71) |
| High energy mechanism (for example but not limited too: roll over RTC, Fall from >2m, Gun shot wound) | 16 (47.06) |
| Major Trauma is dependant on multiple factors that are unique to the individual patient at a given time | 21 (61.76) |
| Injury Severity Score (>15) | 11 (32.35) |
| Injury causing new neurology | 19 (55.88) |
| Suspected spinal injury requiring immobilisation | 7 (20.59) |
| Suspected abdominal injury causing haemodynamic instability | 26 (76.47) |
| Suspected pelvic injury requiring splinting | 14 (41.18) |
| Other: See below | 1 (2.94) |
| Penetrating trauma, Chest injuries with hypoxia - suspected flail. |  |
| Frailty/age @ a location. Eg. Nursing home v mountain summit |  |
|  |  |

**Table 4. Differences in consensus of individual variables between rounds 1 and 2**

| **Variable identified** | **Consensus (>70%)** | **Round 1 %** | **Round 2 %** |
| --- | --- | --- | --- |
| Life threatening injuries | Yes | 95 | 91 |
| Limb threatening | Yes | 92.5 | 88 |
| Major blood loss | Yes | 87.5 | 91 |
| Suspected abdominal injury with haemodynamic instability | Yes | 80 | 76 |
| Injury causing reduced consciousness | Yes | 72.5 | 65 |
